# Ethylene Is Not Essential for R-Gene Mediated Resistance but Negatively Regulates Moderate Resistance to Some Aphids in *Medicago truncatula*

**DOI:** 10.3390/ijms21134657

**Published:** 2020-06-30

**Authors:** Lijun Zhang, Lars G. Kamphuis, Yanqiong Guo, Silke Jacques, Karam B. Singh, Ling-Ling Gao

**Affiliations:** 1 CSIRO Agriculture and Food, Wembley, WA 6014, Australia; butterflycoco@163.com (L.Z.); lars.kamphuis@csiro.au (L.G.K.); guoyq1979@163.com (Y.G.); silke.jacques@curtin.edu.au (S.J.); 2 College of Plant Protection, Shanxi Agricultural University, Taigu 030801, China; 3 The UWA Institute of Agriculture, The University of Western Australia, Crawley, WA 6009, Australia

**Keywords:** disease resistance, plant defense, herbivore, phytohormone, plant biotic stress, plant signalling, *Medicago truncatula*

## Abstract

Ethylene is important for plant responses to environmental factors. However, little is known about its role in aphid resistance. Several types of genetic resistance against multiple aphid species, including both moderate and strong resistance mediated by R genes, have been identified in *Medicago truncatula*. To investigate the potential role of ethylene, a *M. truncatula* ethylene- insensitive mutant, *sickle*, was analysed. The *sickle* mutant occurs in the accession A17 that has moderate resistance to *Acyrthosiphon kondoi*, *A. pisum* and *Therioaphis trifolii*. The *sickle* mutant resulted in increased antibiosis-mediated resistance against *A. kondoi* and *T. trifolii* but had no effect on *A. pisum*. When *sickle* was introduced into a genetic background carrying resistance genes, *AKR* (*A. kondoi* resistance), *APR* (*A. pisum* resistance) and *TTR* (*T. trifolii* resistance), it had no effect on the strong aphid resistance mediated by these genes, suggesting that ethylene signaling is not essential for their function. Interestingly, for the moderate aphid resistant accession, the *sickle* mutant delayed leaf senescence following aphid infestation and reduced the plant biomass losses caused by both *A. kondoi* and *T. trifolii*. These results suggest manipulation of the ethylene signaling pathway could provide aphid resistance and enhance plant tolerance against aphid feeding.

## 1. Introduction

Aphids (Hemiptera: Aphidoidea) constitute a large group of sap-sucking insect pests that cause substantial losses to agriculture worldwide by draining plant nutrients and transmitting pathogenic viruses [1,2,3]. The plant-aphid interaction is distinctive from plant interactions with microbial pathogens and chewing insects mainly because aphid infestation instigates very little physical damage to the plant. With their stylets, aphids penetrate plant tissues by piercing intercellularly through epidermal and mesophyll cell layers and ultimately feed specifically from the phloem sieve element [1,2,3,4,5]. A number of plant genes or loci, including Resistance (*R*) genes, that modulate plant defenses against aphids have been identified in a range of plant species against various aphid species ([6,7,8]. Molecular studies have revealed that plant phytohormones are also involved in the regulation of plant interactions with aphids. However, in many cases, the specific roles that phytohormone pathways play in basal and *R* gene-mediated aphid resistance remains largely unknown. 

Ethylene (ET) is a gaseous plant hormone which is involved in the regulation of various developmental as well as abiotic and biotic stress responses. Studies using mutants impaired in ET biosynthesis and signalling demonstrated a direct role for ET in plant defence against microbial pathogens and insect pests and in the control of plant association with beneficial microbes, such as rhizobia and mycorrhizas. ET signalling has also been shown to regulate plant interactions with insect herbivores [4]. 

A number of studies have been conducted to investigate the role that ET plays in both compatible and incompatible plant−aphid interactions. For compatible interactions, most studies were carried out with the generalist aphid *Myzus persicae* (Sulzer) for which there is no natural genetic resistance. However, the results from these studies were inconsistent. Divol et al. [5] and Moran et al. [6]showed that genes involved in ET biosynthesis and signalling were induced in both celery and *Arabidopsis* following infestation by *M. persicae*. Other studies showed that ET accumulation remained unchanged in both *M. persicae*-infested *Arabidopsis* and *Nicotiana attenuate* compared to the un-infested control plants [7,8] When the performance of *M. persicae* was compared between the *Arabidopsis* wild-type and ET-insensitive *etr* or *ein2* mutant plants, Kettles et al. [9]. found that the aphid fecundity did not differ between the *Arabidopsis* wild-type and *etr1* mutant, whilst the *ein2* mutant did show higher *M. persicae* fecundity than wild-type plants. In contrast, Mewis et al. [10] found that the fecundity of both *M. persicae* and *Brevicoryne brassicae* was reduced on the *etr1* mutant compared to wild-type *Arabidopsis* plants. These contradictory results in the *Arabidopsis*−*M. persicae* interaction highlight the need to further study the role of ET in plant-aphid interactions. 

Studies also indicated that ET may modulate *R* gene-mediated plant defence against aphids. In aphid-resistant barley plants, ET production was significantly induced following the infestation by *Schizaphis graminum*, *Rhopalosiphum padi*, and *Diuraphis noxia* [11,12]. Upon feeding by *D. noxia*, transcript levels of ET-related genes increased in aphid resistant wheat plants [13]. Furthermore, the induction of genes involved in ET signalling and downstream responses was also found in both susceptible and resistant interactions of tomato with *Macrosiphum euphorbiae* and in melon with *Aphis gossypii* [14]. However, in melon with *A. gossypii* stronger induction of ET pathway genes was shown in the resistant variety than the susceptible plants, but this was not the case in tomato with *M. euphorbiae*. 

*Medicago truncatula* is a model legume species for studying plant interaction with aphids [15,16]. *M. truncatula* is a host to several important aphid species including *Acyrthosiphon kondoi* (bluegreen aphid), *Therioaphis trifolii* (spotted alfalfa aphid) and *A. pisum* (pea aphid). In *M. truncatula*, various types of resistance against these aphid species have been identified and the resistance is controlled either through major dominant resistance genes and/or quantitative loci [17,18,19,20,21,22,23,24]. In *M. truncatula* cv. Jester, three single dominant resistance genes, named *AKR* (*A. kondoi* resistance), *APR* (*A. pisum* resistance) and *TTR* (*T. trifolii* resistance), provide strong resistance to *A. kondoi*, *A. pisum* and *T. trifolii*, respectively [17,21,22]. In addition to the major dominant resistance genes for *A. kondoi* and *A. pisum*, a second semi-dominant resistance gene termed *AIN* (*Acyrthosiphon induced necrosis*) has been identified [23]. *AIN* confers a moderate level of resistance to both aphid species and forms hypersensitive response (HR-like) necrotic lesions at the site of infestation by both *A. kondoi* and *A. pisum* [23,25]. This locus is present in both Jester and the reference *M. truncatula* accession, Jemalong (A17) which lacks the three major resistance genes, *AKR*, *APR* and *TTR*. The *M. truncatula* cv. Jester is closely related to A17 [21]. When compared with the highly susceptible *M. truncatula* accession A20, A17 shows moderate resistance to all three aphid species [15]. In A17, in addition to the antibiosis resistance conferred by the *AIN* locus, two distinct quantitative trait loci (QTLs) have been identified for tolerance to *A. kondoi* and *A. pisum*, respectively [18]. Furthermore, three QTL involved in the moderate antibiosis and tolerance to *T. trifolii* have also been identified in A17 [20]. 

The molecular mechanisms underlying the various types of aphid resistance in *M. truncatula* are largely unknown. Expression analysis of genes involved in defence signalling pathways indicated that salicylic acid (SA)-related genes were induced in both A17 and Jester following the infestation by *A. kondoi* and *A. pisum* [17,26,27]. However, jasmonic acid (JA)-related genes were highly induced only in Jester when infested by *A. kondoi* suggesting that JA might be involved in the *AKR*-mediated resistance to *A. kondoi*. In the interactions between *M. truncatula* and European biotypes of *A. pisum*, the induction of phytohormones in *Medicago truncatula* was dependent upon the genotypes of both plant and insect as well the time post-infestation and aphid density [28]. There was some induction of hormones in the compatible interaction but higher concentration of JA, SA and medicarpin exhibited during the incompatible interaction. Although Gao et al. [26] showed an induction of ET associated genes following *A. kondoi* infestation of both Jester and A17, little is known about the role of the ET signalling pathway in moderate or *R-*gene mediated responses in *M. truncatula* following aphid predation. 

The primary aim of this study was to determine the role of ET signalling in the different modes of resistance in *M. truncatula* against the three aphid species, *A. kondoi*, *A. pisum* and *T. trifolii*. The ET insensitive mutant *sickle* in the A17 background, provides a useful genetic tool to decipher the function of ET signalling in the control of different plant-aphid interactions [16,29,30]. Therefore, the role of ET in the moderate resistance to aphids found in A17[18,20,23], was tested by comparing aphid performance and plant tolerance in *sickle* to A17 wild-type plants. To examine the role of *R* gene mediated resistance, crosses were made between Jester, which harbours the major resistance genes and *sickle.* Offspring that carry both the homozygous *sickle* mutation and homozygous resistance genes for the respective aphid species were then tested for aphid performance and plant damage caused by aphid feeding. We found that ET is a negative regulator of moderate resistance to *A. kondoi* and *T. trifolii* but not to the Australian, *A. pisum* biotype. Our results also showed that ET is not essential for *R* gene mediated resistance against the three aphid species or for the *AIN* mediated HR-like response to both *A. kondoi* and *A. pisum*. 

## 2. Results

### 2.1. The M. truncatula Sickle Mutant Modulates Aphid Resistance

**Figure 1:**
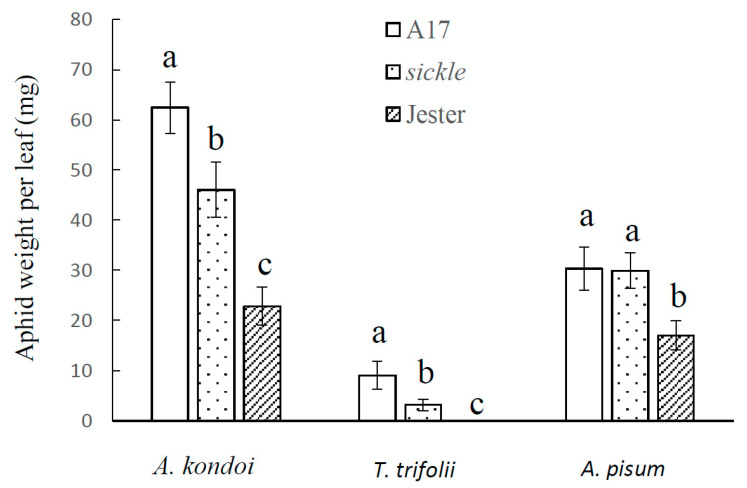
Aphid performance on single intact trifoliate leaves of *Medicago truncatula* genotypes A17, *sickle* and Jester. The aphid performance was shown as aphid fresh weight per trifoliate leaf at seven days following infestation with *Acyrthosiphon kondoi* (five aphids), *Therioaphis trifolii* (seven aphids) and *A. pisum* (four aphids). The values depict the mean and standard error of six biological replicates. The means were only compared among *M. truncatula* genotypes within each aphid species and means with different letters indicate the differences are significant as determined by ANOVA, GenStat (*p* < 0.05).

**Figure 2:**
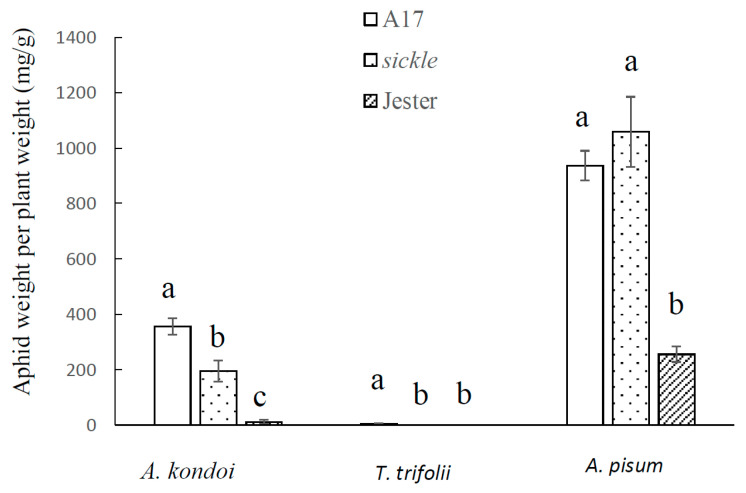
Aphid performance on *Medicago truncatula* genotypes A17, *sickle* and Jester with whole plant assays. The aphid performance was shown as aphid fresh weight per plant dry weight at 14 days following infestation with *Acyrthosiphon kondoi* (five aphids), *Therioaphis trifolii* (seven aphids) and *A. pisum* (four aphids). The values depict the mean and standard error of six biological replicates. The means were only compared among *M. truncatula* genotypes within each aphid species and means with different letters indicate the differences are significant as determined by ANOVA, GenStat (*p* < 0.05).

**Figure 3:**
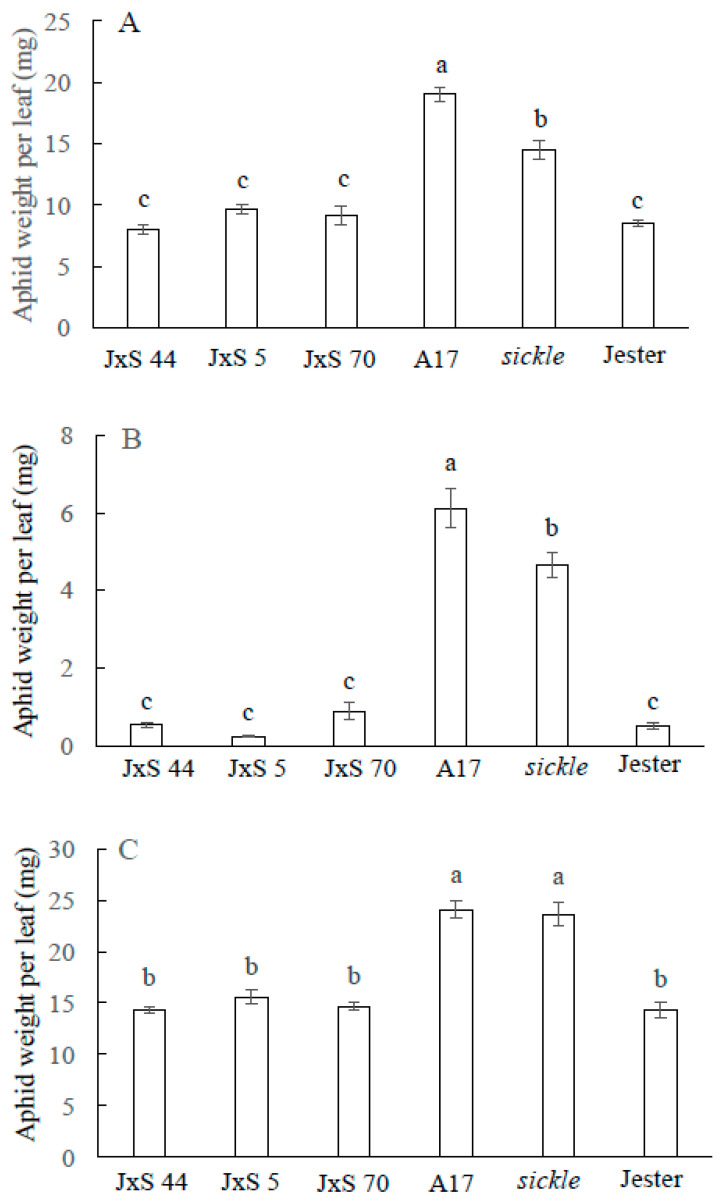
Aphid performance on *Medicago truncatula* accession A17, *sickle*, Jester and three independent F3 lines of Jester x *sickle* crosses, JxS 44, JxS 5 and JxS 70, containing the homozygous *sickle* mutation and all three aphid resistance genes, *AKR* (*Acyrthosiphon kondoi* resistance), *APR* (*Acyrthosiphon pisum* resistance) and *TTR* (*Therioaphis trifolii* resistance), seven days after the infestation by *A. kondoi* (five aphids) (**A**), *T. trifolii* (seven aphids) (**B**) and *A. pisum* (four aphids) (**C**). The values depict the mean and standard error of six biological replicates. For each aphid species, different letters indicate significant differences in the aphid weight between *M. truncatula* genotypes as determined by ANOVA, GenStat (*p* < 0.05).

To examine if the ethylene-insensitive, *M. truncatula sickle* mutant affects the moderate aphid resistance observed in A17, the performance of three aphid species, *A. kondoi*, *T. trifolii* and *A. pisum* was measured. The highly resistant accession, Jester, which carries single dominant resistance genes to all three aphid species and is near isogenic to A17, was included and aphid performance was measured with single trifoliate leaves and subsequently with whole plant assays. Although the durations of aphid infestation were different, seven days in single leaf experiments and 14 days in the whole plant assays, for each aphid species and each *M. truncatula* accession, the results were consistent between these two experiments. As shown in Figure 1 and Figure 2, with all three aphid species, the aphid population weight were significantly higher on the moderately resistant A17 than the highly resistant Jester, which was consistent with our previous reports [17,21,31]. Interestingly, for both *A. kondoi* and *T. trifolii*, the aphid weight was significantly lower (*p* < 0.05) on *sickle* than its wild-type parent, A17, but significantly higher (*p* < 0.05) than on Jester plants. The reduction of aphid population on the *sickle* plants was most pronounced for *T. trifolii* (Figure 1 and Figure 3). At seven days following aphid infestation on single trifoliate leaves, the average aphid population weight per trifoliate leaf on *sickle* was reduced to one third that on A17. In the whole plant experiments, on A17, aphids were able to feed but performed poorly with low aphid weight per plant dry weight (2.36 mg/g) (Figure 2). In contrast, no aphids were observed on *sickle* or on Jester plants after 14 days of aphid infestation. However, for *A. pisum*, the aphid weight did not differ significantly between *sickle* and A17 but was significantly higher than on Jester. Consistent results were obtained in repeat experiments. 

### 2.2. Effect of M. truncatula Sickle Mutant on Plant Tolerance to Aphid Feeding

**Figure 4:**
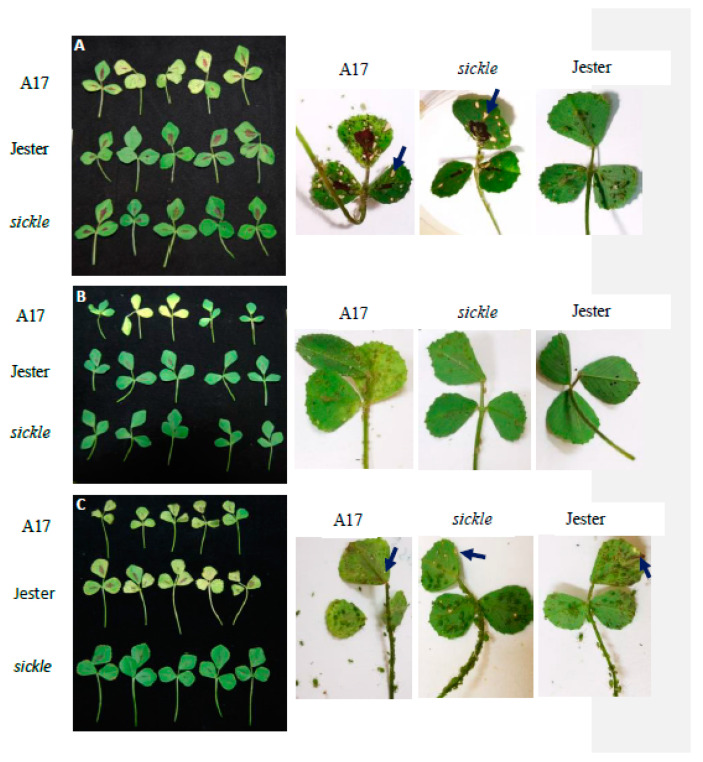
The damage symptoms on single intact trifoliate leaves of *Medicago truncatula* genotypes A17, *sickle* and Jester (left panels). The necrotic flecks (indicated by arrows) on each *M. truncatula* genotype are depicted on the right. The photos were taken at seven days following infestation with *Acyrthosiphon kondoi* (five aphids) (**A**), *Therioaphis trifolii* (seven aphids) (**B**) and *A. pisum* (four aphids) (**C**).

Prior to aphid infestation, the leaves of A17, *sickle* and Jester plants (three weeks after planting) showed no noticeable phenotypical difference. Experiments with single trifoliate leaves demonstrated that following aphid feeding for seven days all three aphid species caused significant damage to the leaves of A17, resulting in reduced leaf size and leaf senescence (Figure 4). The infestation of *A. kondoi* and *A. pisum* also caused leaf senescence on the resistant Jester plants despite much lower aphid population weight on Jester than on A17. Interestingly, with all three aphid species, the leaf senescence was not noticeable on the leaves of *sickle*, even though higher aphid populations were observed on *sickle* than on the resistant Jester plants. This was most apparent with *A. pisum* where on *sickle* leaves the aphid population levels were comparable to A17, and significantly higher than on Jester (Figure 1 and Figure 4). Consistent outcomes were obtained under both growth cabinet and glasshouse conditions in three repeat experiments. 

**Figure 5:**
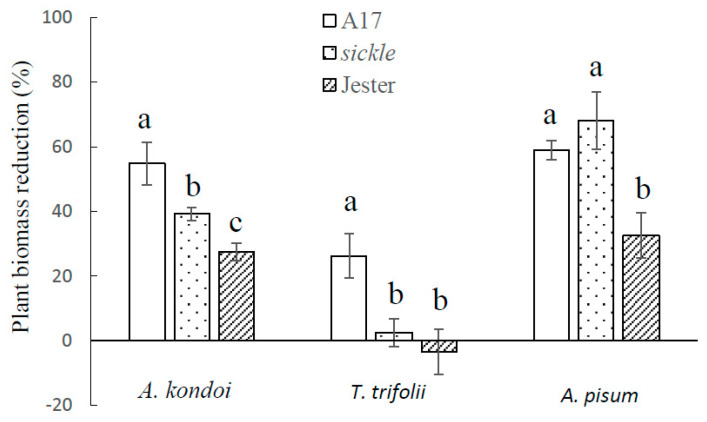
Plant tolerance of *Medicago truncatula* genotypes A17, *sickle* and Jester with whole plant assays. The plant tolerance was measured as the percentage of plant biomass reduction caused by the infestation of *Acyrthosiphon kondoi* (five aphids), *Therioaphis trifolii* (seven aphids) and *A. pisum* (four aphids) for 14 days. The values depict the mean and standard error of six biological replicates. The means were only compared among *M. truncatula* genotypes within each aphid species and means with different letters indicate the differences are significant as determined by ANOVA, GenStat (*p* < 0.05).

The tolerance responses of *sickle*, A17 and Jester plants to aphids when measured with whole plants were consistent with the results on single trifoliate leaves. As illustrated in Figure 5, for each aphid species, the degree of plant biomass reduction caused by aphid infestation correlated with the aphid population levels shown in Figure 2. A17 showed the highest plant biomass reduction relative to the un-infested control plants. In response to the infestation by both *A. kondoi* and *T. trifolii*, *sickle* demonstrated significantly (*p* < 0.05) lower plant biomass reduction than A17. With *A. kondoi*, the biomass reduction in *sickle* was significantly (*p* < 0.05) higher than Jester; however, with *T. trifolii*, the plant biomass of *sickle* or Jester was not significantly different between aphid-infested and control plants. In contrast, the plant biomass reduction caused by *A. pisum* infestation did not differ between *sickle* and A17, which was significantly (*p* < 0.05) higher than that of Jester (Figure 5).

### 2.3. Effect of M. truncatula Sickle Mutant on R Gene Mediated Resistance to Aphids

To examine if the *sickle* mutation affects *R* gene mediated aphid resistance, we crossed the *sickle* locus (located on chromosome 7) to the Jester background. Jester contains all three single dominant aphid resistance genes *AKR*, *APR* and *TTR*, which are closely linked and located on chromosome 3 [17,22,32,33]. The F_2_ plants from these crosses were first screened for the *sickle* locus using 1-aminocyclopropane-1-carboxylic acid (ACC) (see Materials and Methods). Out of 512 F_2_ seedlings screened, 163 showed normal embryonic root growth similar to ACC treated *sickle* mutants and untreated controls, which is consistent with a 1:3 segregation ratio (Chi-square = 2.34, *p* = 0.125) for the 163 lines containing the homozygous *sickle* allele. A subset (90) of 163 pre-selected *sickle* mutant lines were further assessed using high throughput, Multiplex-Ready marker technology and molecular markers linked to these resistance gene loci. Nine homozygous *sickle* plants also contained homozyogous alleles of all three aphid resistance genes (Supplementary Table S2).

To investigate if the *sickle* mutation affects *R* gene mediated resistance to the three aphid species, aphid performance and leaf tolerance were first measured on single trifoliate leaves of individual F_2_ plants with the *sickle* mutation and the three aphid resistance loci and the results compared to *sickle*, A17 and Jester. A follow-up experiment with three randomly selected F_3_ lines, each containing homozygous *sickle*, *AKR*, *APR* and *TTR* alleles was conducted. The results were consistent in both studies using F_2_ or F_3_ plants, but only the results using the F_3_ homozygous lines are presented in Figure 3. With all three aphid species, aphid weights on the control plants of *sickle*, A17 and Jester were consistent to the results shown in Figure 1. For each aphid species, the aphid weights on the three independent F_3_ lines were not significantly different (*p* > 0.05) from each other and did not differ significantly from the aphid weight on the Jester plants (Figure 3). The results demonstrate that the ET insensitive *sickle* mutation has no impact on the antibiosis effect conferred by the three aphid *R* genes; *AKR*, *TTR* and *APR*, against *A. kondoi*, *T. trifolii* and *A. pisum*, respectively.

### 2.4. The Role of Ethylene Insensitivity in the AIN-Mediated Hypersensitive Response to A. kondoi and A. pisum Infestation 

**Figure 6:**
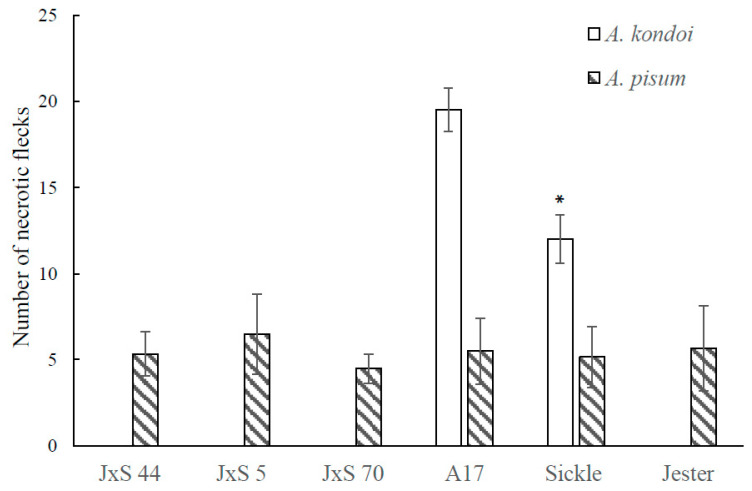
The numbers of necrotic flecks on *Medicago truncatula* accessions A17, *sickle*, Jester and three independent F3 lines of Jester x *sickle* crosses, JxS 44, JxS 5 and JxS 70, containing the homozygous *sickle* mutation and all three aphid resistance genes, *AKR* (*Acyrthosiphon kondoi* resistance) and *APR* (*A. pisum* resistance), seven days after the infestation by *A. kondoi* (five aphids) and *A. pisum* (four aphids). The values depict the mean and standard error of six biological replicates. The means were only compared among *M. truncatula* accessions within each aphid species. The differences in the number of necrotic flecks caused by *A. pisum* are not significant as determined by ANOVA, GenStat (*p* > 0.05). With *A. kondoi*, the necrotic flecks were not observed on *M. truncatula* accession, Jester, JxS 44, JxS 5 and JxS 70, containing homozygous *sickle* and *AKR* alleles. * indicates that with *A. kondoi* the numbers of necrotic flecks on A17 and *sickle* are significantly different as determined by ANOVA, GenStat (*p* < 0.05).

Both *M. truncatula* A17 and Jester carry the semi-dominant *AIN* gene (*Acyrthosiphon*-induced necrosis) which causes HR-like necrotic flecks upon feeding by both *A. kondoi* and *A. pisum* [23]. To evaluate if the *sickle* mutant interacts with AIN-mediated necrosis, the number of necrotic flecks per trifoliate leaf were recorded. *A. kondoi* and *A. pisum* both induced necrotic like spots on A17 and *sickle* (Figure 4 and Figure 6). As shown in Figure 4, when infested with *A. kondoi*, the average number of necrotic flecks per leaf varied significantly (*p* < 0.05) between A17 and *sickle* with 19.5 and 12 per leaf, respectively (Figure 6). In contrast, upon feeding by *A. kondoi* there were no macroscopic lesions observed on Jester nor on leaves of F_2_ and F_3_ lines containing both homozygous *sickle* and *AKR* loci (Figure 4 and Figure 6). When infested with *A. pisum*, necrotic spots were observed on the leaves of all *M. truncatula* accessions examined. The number of necrotic spots per leaf did not differ significantly (*p* > 0.05) between A17 and *sickle* or between Jester and lines carrying both the *sickle* and *APR* loci (Figure 6). 

## 3. Discussion

The model legume *M. truncatula* provides a great opportunity to decipher the molecular mechanisms underlying plant defence against sap-sucking insects, where various types of interactions have been identified to multiple aphid species [16]. The well characterised, ET insensitive, *M. truncatula sickle* mutant allowed us to determine the specific roles that ET plays in plant-aphid interactions. We have found that the *sickle* mutant enhanced the antibiosis effect on *A. kondoi* and *T. trifolii* but not *A. pisum* (Figure 1 and Figure 2), and delayed leaf senescence caused by the feeding of all three aphid species. We show that the ET signalling pathway is not essential for the function of the major aphid resistance genes, *AKR*, *APR* or *TTR* against the three aphid species and is also not required for the *AIN*-mediated hypersensitive response to *A. kondoi* or *A. pisum* infestation. 

Our results showed that on *sickle*, the growth of both *A. kondoi* and *T. trifolii* colonies was significantly reduced compared to its wild-type parent, A17. The results suggest that ET is a negative regulator of the moderate resistance in *M. truncatula* against these two aphid species. The ET signalling pathway has previously been demonstrated as a negative regulator in other plant species against sap-sucking insects. For instance, in rice, the suppression of ET biosynthesis enhanced resistance against a piercing-sucking insect, the brown planthopper (*N. lugens*) but reduced plant resistance against a chewing insect, striped stem borer (*C. suppressalis*) [34]. In *Arabidopsis*, several studies suggested that ET is a negative regulator of aphid defense responses. The fecundities of the generalist *M. persicae* and the specialist *Brevicoryne brassicae* were reduced on the ET-insensitive *etr1* mutant compared to wild-type plants [10,35]. The overexpression of a transcription factor gene, *MYB102*, which promotes ET biosynthesis by upregulation of some 1-aminocyclopropane-1-carboxylate synthase (ACS) genes in the ET-synthetic pathway led to an increase in aphid performance [36]. Furthermore, in tomato, ET signalling contributes to the susceptibility of potato aphid, *M. euphorbiae*, in the absence of the *Mi-1.2* gene. In choice assays, potato aphids preferred wild-type plants to the ET-insensitive, *Neverripe* mutant [37]. Our results with *sickle* together with other results discussed, suggest that ET can benefit the feeding for some aphid species, and the impediment of ET pathway impairs the infestation of these aphids. 

In contrast to *A. kondoi* and *T. trifolii*, the *sickle* mutant did not affect the growth of the Australian biotype of *A. pisum* (Figure 1 and Figure 2). These findings were consistent with studies with the European *A. pisum* biotype (PS01), which is distinct from the Australian *A. pisum* biotype, and where ET was also found to not be involved in the aphid susceptible or resistant interactions [24,28]. However, *sickle* was found to promote the growth of a Chinese biotype of *A. pisum* [38,39,40]. These differences between the *A. pisum* biotypes and with *A. kondoi* and *T. trifolii* suggest that the role of ethylene in the *M. truncatula*-aphid interactions is both biotype- and species-dependent. 

How ET signalling modulates the moderate resistance in A17 against *A. kondoi* and *T. trifolii* is unknown. A17 carries multiple QTLs conferring antibiosis factors against these two aphid species [18,20]. The direct link between the ET signalling pathway and a specific QTL(s) in A17 is yet to be investigated. It is possible that the suppression of the ET pathway in *sickle* led to upregulation of other signalling pathways, such as for SA and JA, which might increase plant defence mechanisms against these two aphid species, as these signalling pathways are often inter-linked and work synergistically or antagonistically [41,42,43]. Further research on the interactions between the ET insensitivity in *M. truncatula sickle* and other defense signalling pathways in plant-aphid interactions will facilitate the understanding of the function of ET in plant resistance to aphids. 

Our results also showed that the *sickle* mutant delayed the leaf senescence caused by the feeding of all three aphid species. There was no noticeable phenotypical difference among the leaves of A17, *sickle* and Jester control plants prior to aphid infestation at three weeks after planting though *sickle* could demonstrate concomitant alternation of some ethylene related phenotypes, including delayed petal and leaf senescence and decreased abscission of seed pod and leaves at the later stage of plant growth [29]. As all our experiments were carried out with young plants of three to five weeks old, it is unlikely these concomitant ethylene related phenotypes have a direct impact on aphid performance or plant symptom in response to aphid infestation. Aphid infestation causes changes in source allocation in the host plant to direct nutrients to the insect infested tissues [44]. Premature leaf senescence has been suggested to be a plant defence mechanism used to counteract aphid feeding by redirecting the nutrients to the un-infested source tissues [45]. In *Arabidopsis,* infestation by *M. persicae* induced the transcription level of *SENESCENCE ASSOCIATED GENES* (*SAG*s). Silencing of the *SAG*s delayed plant senescence which led to an increase *M. persicae* levels [45]. Here we observed the opposite with the *M. truncatula sickle* mutant. Whether the delay in leaf senescence directly relates to the increased antibiosis resistance to *A. kondoi* and *T. trifolii* remains unknown. Further research would help elucidate the relationship between leaf senescence and aphid feeding processes. 

We have determined that ET is not essential for *AKR*, *APR* or *TTR* mediated resistance against *A. kondoi*, *A. pisum* or *T. trifolii*, respectively. ET has also been found to be dispensable for the *RAP* gene mediated resistance against the European *A. pisum* biotype [28]. However, upon infestation by *A. kondoi* or *T. trifolii*, some ET related genes were found to be induced in both A17 and Jester, with higher induction in Jester than A17 [26,27]. The lack of difference in aphid performance between Jester and Jester with the homozygous *sickle* mutation suggests that induction of the ET related genes previously found in Jester may be insufficient in limiting aphid feeding from the plant [26]. This might also be the case in other plant-aphid systems, such as in resistant barley plants with *Schizaphis graminum*, *Rhopalosiphum padi*, and *Diuraphis noxia* [11,12], wheat with *D. noxia* [13], tomato (*Mi1.2*) with *Macrosiphum euphorbiae* and melon (*Vat*) with *Aphis gossypii* [14]. Although in these plant-aphid systems, ET production or ET related genes were shown to be highly induced in the resistant interactions, whether ET contributes to the resistance outcome is still a question. 

Both A17 and Jester carry a semi-dominant locus called *AIN* which mediates necrotic lesions resembling a hypersensitive response at the site of infestation by both *A. kondoi* and *A. pisum* [23]. It is unlikely that ET signalling is negatively regulating the activity of *AIN* in A17 for the following reasons: firstly, while *AIN* is important for resistance to both *A. kondoi* and *A. pisum* in A17, the *sickle* mutant only results in an increase in resistance *to A. kondoi*. Secondly, the *sickle* mutant still displays the same HR-like symptoms conferred by *AIN* following infestation with either aphid. While the overall number of necrotic lesions were less in *sickle* than A17 following *A. kondoi* infestation, this is most likely a reflection of the lower number of aphids feeding on *sickle*, due to the increase in aphid resistance. Importantly, the size of the necrotic lesions remained similar between *sickle* and A17. Collectively, these data suggest that ET signalling is not a negative regulator of *AIN* activity. As discussed earlier, there are other QTLs that have been identified in A17 as being important for the moderate resistance to *A. kondoi* but not *A. pisum* [18], which may be the target of ET negative regulation, or the target(s) may be an unidentified loci.

In conclusion, the *M. truncatula sickle* mutant has previously been shown to be defective in the control of root infecting micro-organisms including beneficial rhizobia, mycorrhizal fungi, as well as infection by the fungal and oomycete pathogens, *R. solani* and *P. medicaginis* [46,47]. Here we observed a positive effect of the *sickle* mutant on the control of the infestation by insect herbivory, by *A. kondoi* and *T. trifolii*. While ET signaling is not essential for the activity of three R genes for resistance against *A. kondoi*, *A. pisum* and *T. trifolii,* it is also not involved in the *AIN* mediated hypersensitive response to *A. kondoi* and *A. pisum*. However, the *sickle* mutant delayed leaf senescence by all three aphid species, but enhanced tolerance only to infestation by *A. kondoi* and *T. trifolii*, The results suggest that manipulation of the ET signaling pathway could also help provide resistance to certain aphid species and enhance plant tolerance against aphid feeding.

## 4. Materials and Methods

### 4.1. Plant Materials and Growth Conditions

Included in this study were *M. truncatula* A17 (referred to as “wild-type”), which is the reference *M. truncatula* accession, an ethylene insensitive *sickle* mutant, a *tnt1* retrotransposon mutant that arose from a single *tnt1* insertion in the genetic background of A17 [29,30] and Jester which is closely related to A17 sharing 89% genome identity [21]. In addition to ethylene insensitivity, *sickle* also demonstrates delayed petal senescence and decreased abscission of seed pod and leaves. In addition F_2_ and F_3_ progenies were generated by reciprocal crossing between Jester and *sickle* accession plants according to the methods described by [48]. 

Prior to planting, seeds were scarified and germinated on moist filter paper in the dark at room temperature for two days. Plants were grown in a growth chamber with 16 h light (22 °C)/8 h dark (20 °C) under metal halide and incandescent lamps producing 240 to 260 µE m^−2^ s^−1^ or in a glasshouse with controlled temperature around 22 °C and ambient light condition. In both glasshouse and growth chamber experiments, plants were grown in individual 0.9 L pots with *Arabidopsis* soil mix (Richgrow company, Perth, WA, Australia). Plants were fertilized with liquid Nitrosol fertilizer (Amgrow Australia, Perth, WA, Australia) once planted and watered two times per week throughout the experiments. 

### 4.2. Aphid Species and Rearing Conditions 

The aphid species used were *A. kondoi* (bluegreen aphid), *A. pisum* (pea aphid.) and *T. trifolii* f. *maculate* (spotted alfalfa aphid). Aphids of each species were obtained from colonies initiated from single aphid clones collected in Western Australia and were reared on Subterranean clover (*Trifolium subterraneum*) for *A. kondoi*, faba bean (*Vicia faba* L.) for *A. pisum* and alfalfa (*M. sativa*) for *T. trifolii* with 14 h light (23 °C)/10 h dark (20 °C) under high pressure sodium lamps and fluorescent light at 280 µE m^–2^ s^–1^. Aphids were transferred to experimental plants with a fine paintbrush. 

### 4.3. Screening for F_2_ Plants Containing the Sickle Homozygous Allele

To obtain lines with the homozygous *sickle* locus and all three aphid resistance genes, *AKR*, *APR* and *TTR* crosses between *sickle* and Jester were performed F_2_ plants were first screened for the *sickle* mutation using 1-aminocyclopropane-1-carboxylic acid (ACC) [30]. To establish an effective and reliable condition for the screening, five concentrations of ACC, 20, 40, 60, 80 and 100 ppm were initially evaluated with the *sickle* mutant, A17 and Jester with water as the control. While the *sickle* mutant showed no response to all ACC concentrations, 20 ppm ACC started to show impact on the embryonic root of A17 and Jester, while 80 ppm or 100 ppm of ACC resulted in severe stunting of root radicles of both A17 and Jester (Supplementary Figure S1). The 100 ppm was used for the screening of the Jester x *sickle* F_2_ population (Supplementary Figure S2).

### 4.4. Genotyping F_2_ Plants with AKR, APR and TTR Loci

In order to obtain F_2_ lines that combined the homozygous *sickle* allele and the three aphid resistance gene loci, 90 of the 163 pre-selected F_2_ *sickle* mutant plants were randomly selected and analysed using high throughput Multiplex-Ready marker technology (MRT) and molecular markers linked to these loci (Supplementary Table S1). The plants were grown in the growth room conditions as described above. Two weeks after planting, a single trifoliate leaf from each plant was collected and DNA isolated using the CTAB method as described previously [49]. DNA was subsequently diluted to a concentration of 50 ng/µL in a 96-well plate and multiplex ready PCRs were setup using the primers in Supplementary Table S1 and the protocol described by Hayden et al. [50]. The multiplexed PCR products were subjected to fragment analysis on an ABI3730 DNA analyser (Applied Biosystems, Melbourne, Victoria, Australia) according to Hayden et al. [50] and marker allele sizing determined using the Genemarker software (SoftGenetics LLC, State College, PA, USA).. After the genotyping, seeds from the F_2_ plants with both the homozygous *sickle* and the three homozygous aphid resistance gene alleles, *AKR*, *APR* and *TTR*, were harvested to obtain F_3_ seeds for the subsequent aphid infestation experiments. 

### 4.5. Aphid Performance and Plant Damage on Single Trifoliate Leaves

To assess the aphid performance and leaf tolerance of *sickle* in comparison with its wild-type, parent A17 and Jester against three aphid species, three experiments were conducted, one in the glasshouse and two in growth chambers. Six replicate plants of each *M. truncatula* accession were randomly arranged. For all three experiments, three weeks after sowing, a single trifoliate leaf of similar age (fourth or fifth trifoliate leaf to emerge on the primary stem) of each plant was infested with four, five or seven adults of *A. pisum*, *A. kondoi* or *T. trifolii*, respectively. The number for each aphid species was determined based on our previous experiences with regards to the aphid size, the speed of aphid reproduction and degree of leaf damage caused to create a condition that allowed the aphid growth and leaf damage to be fully expressed to make comparison between the three *M. truncatula* accessions [17,22,23,26,31,32,33]. The aphids were caged on a single trifoliate leaf in a linen mesh cage (35 × 200 mm) per plant. A wooden stake supported the stem and cage [26]. Seven days after aphid infestation, the aphids on each leaf were collected and weighed. The damage on each leaf was visually assessed after the removal of the aphids.

The effect of the *sickle* mutant on *R* gene mediated aphid resistance was first measured using the F_2_ plants after the genotyping. This was followed by an experiment using three independent F_3_ lines which contained the *sickle*, *AKR*, *APR* and *TTR* homozygous alleles. A17, Jester and the *sickle* mutant were included for comparison. For each accession/F3 line, six replicate plants were set up for the aphid infestation as described above with single trifoliate leaves. The aphid population weight and leaf damage symptoms, such as leaf senescence and necrosis, were assessed. To examine if the *sickle* mutant affects the *AIN* -mediated hypersensitive response to *A. kondoi* and *A. pisum*, the numbers of macroscopic necrotic flecks per single trifoliate leaves were also recorded. 

### 4.6. Aphid Performance and Plant Tolerance Experiments on Whole Plants

To assess the aphid performance and plant tolerance on whole plants, non-choice experiments were conducted under glasshouse conditions. Plants of *M. truncatula sickle* mutant, A17 and Jester were grown as described above. Two weeks after planting, each plant was infested with four, five or seven adults of *A. pisum*, *A. kondoi* or *T. trifolii*, respectively. Six replicate plants were set up for each *M. truncatula* accession with or without aphid infestation. Plants were randomly arranged. Fourteen days after the aphid infestation, aphids were collected from each plant and weighed immediately. After the removal of the aphids, the aerial part of all the plants including the non-infested control plants were dried in the oven at 50°C for two days. The dried weight of each plant was recorded. For each plant, aphid fresh weight per plant dry weight was calculated to determine aphid performance on the plant. For each *M. truncatula* accession, the tolerance of individual plants to aphid infestation was measured as the percentage of plant biomass reduction (PBR) relative to mean biomass of the control plants of the same *M. truncatula* accession using the formula: PBR = [(A − B)/A] × 100, in which A: average of the non-infested plant dry weight; B: dry weight of individual aphid-infested plant. 

With each experiment, the aphid weight, the number of necrotic flecks or plant biomass reduction were analyzed by one-way ANOVA and compared by the LSD test at a 5% significance level using GenStat (VSN International, Rothamsted Research, Hertfordshire, UK). 

## Supplementary Materials

The following are available online at https://www.mdpi.com/1422-0067/21/13/4657/s1. Table S1. Overview of the PCR primers used; Table S2. Overview of the genotyping data. Supplementary Figure S1. The radicle root growth of *Medicago truncatula* A17, Jester and *sickle* mutant. Supplementary Figure S2. Screening of *Medicago truncatula* F_2_ plants of crosses between Jester and *sickle*.
