# Supplementary material for: Ethylene Is Not Essential for R-Gene Mediated Resistance but Negatively Regulates Moderate Resistance to Some Aphids in Medicago truncatula
Source: Int J Mol Sci. 2020 Jun 30;21(13):4657. doi: 10.3390/ijms21134657 (PMC7369913; doi:10.3390/ijms21134657)
Supplement: Supplementary file 1 [file ijms-21-04657-s001.zip › ijms-835928-revised-r1-supplementary/Supplementary Figures.docx]

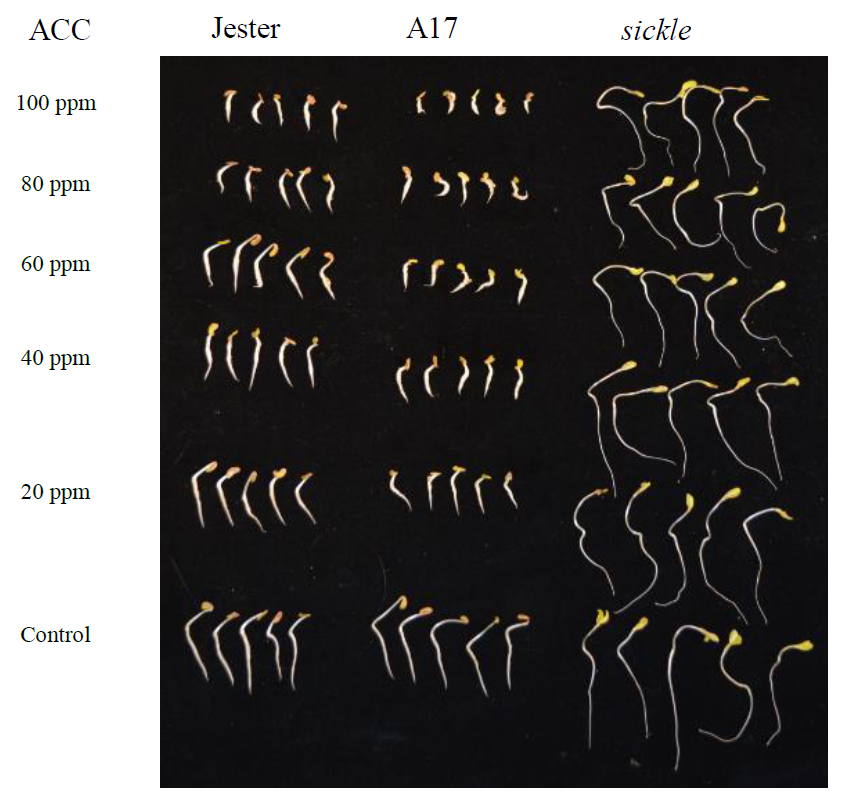


Supplementary Figure S1. The radicle root growth of *Medicago truncatula* A17, Jester and *sickle* mutant three days after treatment with a concentration series of 1-aminocyclopropane-1-carboxylic acid (ACC) or water as control.


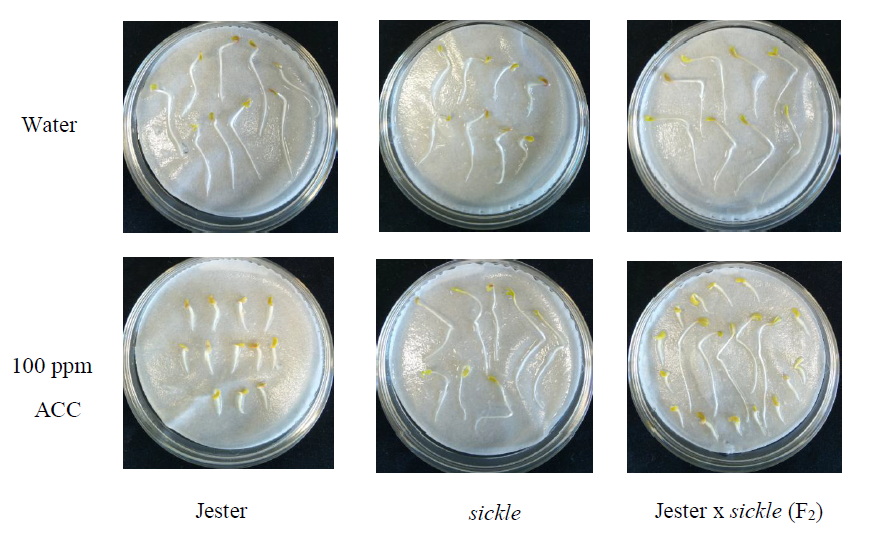


Supplementary Figure S2. Screening of *Medicago truncatula* F_2_ plants of crosses between Jester and *sickle* (in what background?) to recover seedlings with ahomozygous *sickle* mutation. Photos were taken three days after treatment with 100 ppm of 1-aminocyclopropane-1-carboxylic acid (ACC) or water as control. Seedlings carrying the homozygous *sickle* mutation showed no response to ACC whereas seedlings with the wild type or heterozygous allele had stunted root radicles.
