# Supplementary material for: Ethylene Is Not Essential for R-Gene Mediated Resistance but Negatively Regulates Moderate Resistance to Some Aphids in Medicago truncatula
Source: Int J Mol Sci. 2020 Jun 30;21(13):4657. doi: 10.3390/ijms21134657 (PMC7369913; doi:10.3390/ijms21134657)
Supplement: Supplementary file 1 [file ijms-21-04657-s001.zip › ijms-835928-revised-r1-supplementary/Supplementary Table 1.docx]

**Table S1.** Overview of the PCR primers used for the high throughput genotyping of the Jester x *sickle* F_2_ individuals summarised in Supplementary Table 2.

| Locus name | Forward primer | Reverse Primer | Flanking marker for aphid resistance gene |
| --- | --- | --- | --- |
| 30GTT7 | ACGACGTTGTAAAAGAGGGGAAACCTTCTTCGTC | CATTAAGTTCCCATTAAACAGTGAACCGTCAGCACA | *AKR* |
| 593AT3V | ACGACGTTGTAAAACTTCATTCAACAAATGAGCA | CATTAAGTTCCCATTACATATCATCTCATGGGTCCT | *AKR* |
| 004H01 | ACGACGTTGTAAAATCCCAATTTATGTTGTCCTC | CATTAAGTTCCCATTATTGTTATGAAGGGGAAGAGA | *AKR* |
| T38L12-21 | ACGACGTTGTAAAACTTTTGTGTTATGGGAGGAACC | CATTAAGTTCCCATTATGGCATTTTCGTCATAGGC | *TTR* |
| T38L12-100 | ACGACGTTGTAAAATGGGTTATCCGGTCCTAACG | CATTAAGTTCCCATTAGTCCACATCACCAATTTTACGG | *TTR* |
| TT130+AGGA | ACGACGTTGTAAAACCAAGGTAAGTGACAGGTCTGC | CATTAAGTTCCCATTAGACGGTGGACGATGATGG | *TTR* |
| TT132+TG | ACGACGTTGTAAAATATCAGCTGCAACAATGATGG | CATTAAGTTCCCATTAAGATTGTTAATAACGTTCCTGAAGC | *TTR* |
| LK269/270 | ACGACGTTGTAAAATCGTGGATGTATCTTCTGTCTGA | CATTAAGTTCCCATTAACAACCTCCTCTTTGCCAATAA | *APR* |
| LK315/316 | ACGACGTTGTAAAATGAATGGTCGTATGATTTCTGG | CATTAAGTTCCCATTACCCTTTTCTTGTTTGACTCCAC | *APR* |
| LK273/274 | ACGACGTTGTAAAACTGCATTTAGGTGTGCAATCAT | CATTAAGTTCCCATTACCTTCTTGGGAGAGTTTCAATG | *APR* |
