# Supplementary material for: Ethylene Is Not Essential for R-Gene Mediated Resistance but Negatively Regulates Moderate Resistance to Some Aphids in Medicago truncatula
Source: Int J Mol Sci. 2020 Jun 30;21(13):4657. doi: 10.3390/ijms21134657 (PMC7369913; doi:10.3390/ijms21134657)
Supplement: Supplementary file 1 [file ijms-21-04657-s001.zip › ijms-835928-revised-r1-supplementary/Supplementary Table 2.docx]

| **Table S2.** Overview of the genotyping data that identified nine Jester x *sickle* F_2_ individuals (highlighted in blue) to have the homozygous *sickle* mutation and homozygous alleles for the Jester derived resistance genes *AKR*, *TTR* and *APR*. The molecular markers represent markers that either flank or co-segregate with the aphid resistance phenotype. The alleles derived from the MRT PCRs can be homozygous A17/*sickle* (A = A17/*sickle* allele); Heterozygous with an allele from either parent (H = Heterozygous); or homozygous Jester allele (B = Jester allele). The dash represents no data for the molecular marker for a given individual. | | | | | | | | | | | | |
| --- | --- | --- | --- | --- | --- | --- | --- | --- | --- | --- | --- | --- |
|  | *AKR* region | | |  | *TTR* region | | | |  | *APR* region | | |
| Jester x *sickle* F_2_ Line | 30GTT7 | 593AT3V | 4H01 |  | T38L12-21 | T38L12-100 | TT130+AGGA | TT132+TG |  | LK269/270 | LK315/316 | LK273/274 |
|  |  |  |  |  |  |  |  |  |  |  |  |  |
| Jxsickle-1 | B | B | B |  | - | B | B | B |  | H | A | A |
| Jxsickle-2 | B | B | B |  | H | H | H | H |  | H | H | H |
| Jxsickle-3 | H | H | H |  | H | A | A | A |  | A | A | A |
| Jxsickle-4 | B | B | B |  | H | H | H | H |  | H | H | H |
| Jxsickle-5 | B | B | B |  | B | B | B | B |  | B | B | B |
| Jxsickle-6 | H | H | H |  | A | A | A | A |  | A | A | A |
| Jxsickle-7 | A | A | A |  | A | A | A | A |  | H | H | H |
| Jxsickle-8 | H | A | H |  | H | H | H | H |  | B | B | B |
| Jxsickle-9 | A | A | A |  | H | H | H | H |  | H | H | H |
| Jxsickle-10 | H | H | H |  | B | B | B | B |  | B | B | B |
| Jxsickle-11 | H | H | H |  | H | H | H | H |  | H | H | H |
| Jxsickle-12 | A | A | A |  | A | A | A | A |  | H | H | H |
| Jxsickle-13 | H | H | H |  | A | A | A | A |  | A | A | A |
| Jxsickle-14 | B | B | B |  | H | H | H | H |  | H | H | H |
| Jxsickle-15 | H | H | H |  | H | H | H | H |  | H | H | H |
| Jxsickle-16 | A | H | - |  | - | H | H | H |  | B | B | B |
| Jxsickle-17 | H | H | H |  | H | H | H | H |  | H | H | H |
| Jxsickle-18 | B | B | B |  | B | B | B | B |  | H | H | H |
| Jxsickle-19 | A | A | A |  | A | A | H | H |  | H | H | H |
| Jxsickle-20 | B | B | B |  | - | B | B | B |  | B | B | - |
| Jxsickle-21 | H | H | H |  | H | H | H | H |  | H | H | H |
| Jxsickle-22 | H | H | H |  | A | A | A | A |  | A | A | A |
| Jxsickle-23 | A | A | A |  | H | H | H | H |  | H | H | H |
| Jxsickle-24 | H | H | H |  | H | H | H | H |  | H | H | H |
| Jxsickle-25 | H | H | H |  | A | A | A | A |  | A | A | A |
| Jxsickle-26 | B | B | B |  | H | - | H | H |  | H | H | H |
| Jxsickle-27 | B | B | B |  | B | - | B | B |  | B | B | B |
| Jxsickle-28 | H | H | H |  | H | H | H | H |  | H | H | H |
| Jxsickle-29 | H | H | H |  | A | A | A | A |  | A | A | A |
| Jxsickle-30 | B | B | B |  | B | B | - | B |  | B | B | B |
| Jxsickle-31 | A | A | - |  | - | A | A | - |  | H | H | H |
| Jxsickle-32 | H | H | H |  | H | H | H | H |  | H | H | H |
| Jxsickle-33 | A | A | A |  | A | A | A | A |  | A | A | A |
| Jxsickle-34 | B | B | B |  | B | B | B | B |  | - | - | H |
| Jxsickle-35 | B | B | B |  | B | B | B | B |  | H | H | H |
| Jxsickle-36 | H | H | H |  | B | B | B | B |  | B | B | B |
| Jxsickle-37 | A | A | A |  | A | A | H | H |  | H | H | H |
| Jxsickle-38 | H | H | - |  | - | H | H | - |  | H | H | H |
| Jxsickle-39 | H | - | - |  | - | - | H | - |  | H | H | - |
| Jxsickle-40 | H | H | H |  | H | H | H | H |  | B | B | B |
| Jxsickle-41 | B | B | B |  | B | B | B | B |  | B | - | B |
| Jxsickle-42 | A | A | A |  | A | A | A | A |  | A | A | A |
| Jxsickle-43 | A | A | A |  | A | A | A | A |  | A | A | A |
| Jxsickle-44 | B | B | B |  | B | B | B | B |  | B | B | B |
| Jxsickle-45 | A | A | A |  | A | A | A | A |  | A | A | A |
| Jxsickle-46 | H | H | H |  | H | H | H | H |  | H | H | H |
| Jxsickle-47 | H | H | H |  | H | H | H | H |  | A | A | A |
| Jxsickle-48 | H | H | H |  | H | H | H | H |  | - | - | B |
| Jxsickle-49 | H | A | A |  | A | - | B | A |  | H | H | - |
| Jxsickle-50 | A | A | A |  | A | A | A | A |  | B | B | B |
| Jxsickle-51 | H | H | H |  | H | H | H | H |  | H | H | H |
| Jxsickle-52 | H | H | H |  | H | H | H | H |  | - | - | H |
| Jxsickle-53 | H | H | H |  | H | H | B | B |  | B | B | B |
| Jxsickle-54 | A | - | A |  | A | - | A | A |  | A | A | - |
| Jxsickle-55 | H | H | H |  | H | H | H | H |  | - | - | H |
| Jxsickle-56 | B | B | B |  | B | B | B | B |  | B | B | B |
| Jxsickle-57 | H | H | H |  | H | H | H | H |  | - | - | - |
| Jxsickle-58 | H | H | H |  | H | H | H | H |  | B | B | B |
| Jxsickle-59 | A | A | A |  | A | A | A | A |  | A | A | A |
| Jxsickle-60 | A | A | A |  | A | A | A | A |  | H | H | H |
| Jxsickle-61 | A | A | A |  | A | A | A | A |  | A | A | A |
| Jxsickle-62 | H | - | H |  | H | - | H | H |  | H | H | - |
| Jxsickle-63 | B | B | B |  | B | B | B | B |  | A | A | A |
| Jxsickle-64 | - | H | H |  | B | B | B | B |  | B | - | B |
| Jxsickle-65 | H | H | H |  | B | H | B | B |  | H | H | H |
| Jxsickle-66 | H | H | H |  | H | H | H | H |  | H | H | H |
| Jxsickle-67 | H | H | H |  | A | A | A | A |  | A | A | A |
| Jxsickle-68 | A | A | A |  | A | A | A | A |  | A | A | A |
| Jxsickle-69 | A | A | A |  | A | A | A | A |  | A | A | A |
| Jxsickle-70 | B | B | B |  | B | B | B | B |  | B | B | B |
| Jxsickle-71 | A | A | A |  | H | H | B | B |  | B | B | B |
| Jxsickle-72 | B | B | B |  | B | B | B | B |  | H | H | H |
| Jxsickle-73 | H | A | A |  | H | A | H | H |  | - | - | - |
| Jxsickle-74 | H | H | H |  | H | H | H | H |  | A | A | A |
| Jxsickle-75 | H | H | A |  | H | H | H | H |  | - | H | H |
| Jxsickle-76 | A | A | A |  | A | - | A | A |  | A | A | A |
| Jxsickle-77 | H | H | H |  | A | A | A | A |  | H | H | H |
| Jxsickle-78 | A | A | A |  | H | H | H | H |  | H | H | H |
| Jxsickle-79 | A | A | A |  | A | A | A | A |  | A | A | A |
| Jxsickle-80 | B | B | B |  | B | B | B | B |  | B | B | B |
| Jxsickle-81 | H | H | H |  | H | H | H | H |  | H | H | H |
| Jxsickle-82 | A | A | A |  | H | H | H | H |  | H | H | H |
| Jxsickle-83 | A | A | A |  | A | A | A | A |  | A | - | - |
| Jxsickle-84 | - | - | - |  | A | - | A | H |  | - | - | A |
| Jxsickle-85 | H | H | H |  | H | H | H | H |  | H | H | H |
| Jxsickle-86 | H | H | H |  | A | H | H | H |  | B | B | B |
| Jxsickle-87 | H | H | H |  | H | H | B | B |  | B | B | B |
| Jxsickle-88 | B | B | B |  | B | B | B | B |  | B | B | B |
| Jxsickle-89 | A | A | A |  | A | A | A | A |  | A | A | A |
| Jxsickle-90 | H | - | H |  | H | - | H | H |  | H | H | - |
